# Accelerated diffusion-weighted imaging of the prostate employing echo planar imaging with compressed SENSE based reconstruction

**DOI:** 10.1038/s41598-025-94777-6

**Published:** 2025-03-25

**Authors:** Yannik Christian Layer, Petra Mürtz, Alexander Isaak, Leon Bischoff, Barbara Daria Wichtmann, Christoph Katemann, Kilian Weiss, Julian Luetkens, Claus Christian Pieper

**Affiliations:** 1 Department of Diagnostic and Interventional Radiology, University Hospital Bonn, Bonn, Germany; 2 Philips GmbH Market DACH, Hamburg, Germany

**Keywords:** Prostate, Diffusion weighted MRI, Multiparametric magnetic resonance imaging, Echo planar imaging, Prostate cancer, Prostate, Magnetic resonance imaging

## Abstract

Aim was to evaluate accelerated diffusion-weighted imaging (DWI) of the prostate using echo planar imaging with compressed SENSE based reconstruction (EPICS) and assess its performance in comparison to conventional DWI with parallel imaging. In this single-center, prospective study, 35 men with clinically suspected prostate cancer underwent prostate MRI at 3T. In each patient, two different DWI sequences, one with 3 b-values (b = 100, 400, 800s/mm²) for ADC-calculation and one with b = 1500s/mm², were acquired with conventional SENSE and with EPICS. Quantitative evaluation was done by regions-of-interest (ROIs) analysis of prostate lesions and normal appearing peripheral zones (PZ). Apparent contrast-to-noise (aCNR) and apparent signal-to-noise ratios (aSNR) were calculated. Mean ADC and coefficient of variation (CV) of ADC were compared. For qualitative assessment, artifacts, lesion conspicuity, and overall image quality were rated using a 5-point-Likert-scale (1: nondiagnostic to 5: excellent). Additionally, the Prostate Imaging Reporting and Data System (PIRADS 2.1) was rated for DWI. The average total scan time reduction with EPICS was 43%. Quantitative analysis showed no significant differences between conventional SENSE and EPICS, neither for aSNR_Lesion_ (e.g. b1500_conv_: 24.37 ± 10.28 vs. b1500_EPICS_: 24.08 ± 12.2; *p* = 0.98) and aCNR_Lesion_ (e.g. b1500_conv_:9.53 ± 7.22 vs. b1500_EPICS_:8.88 ± 6.16; *p* = 0.55) nor for aSNR_PZ_ (e.g. b1500_conv_:15.18 ± 6.48 vs. b1500_EPICS_: 15 ± 7.4; *p* = 0.94). Rating of artifacts, lesion conspicuity, overall image quality and PIRADS-scores yielded comparable results for the two techniques (e.g. lesion conspicuity for ADC_conv_: 4(2–5) vs. ADC_EPICS_ 4(2–5); *p* = 0.99 and for b1500_conv_: 4(2–5) vs. b1500_EPICS_ 4(2–5); *p* = 0.25). Overall, accelerated DWI of the prostate using EPICS significantly reduced acquisition time without compromising image quality compared to conventional DWI.

## Introduction

Multiparametric MRI of the prostate has become diagnostic standard for evaluation of prostate lesions^1,2^. The combination of T2-weighted images with diffusion-weighted imaging (DWI) and dynamic contrast-enhanced sequences has proven to achieve high sensitivity for the detection of prostate cancer^3,4^. Regarding structured image reporting of the prostate the Prostate Imaging Reporting and Data System (PIRADS) is the generally recognized standard^5^. DWI is the dominant sequence for the assessment of the peripheral zone, where most carcinomas of the prostate are located^6,7^. As the number of patients with prostate cancer increases due to ageing societies and prostate MRI has proven a valuable diagnostic tool in the management of prostate cancer, there is a strong need for dependable but accelerated MRI protocols^8,9^.

Several technologies have been demonstrated to shorten the acquisition times in MRI such as fast spin echo (FSE) imaging^10^, echo planar imaging (EPI)^11^ and fast low angle shot (FLASH)^12^ imaging. One possible approach is the undersampling of k-space, which is used in parallel imaging and compressed sensing (CS)^13^. CS allows for an image reconstruction from a limited number of k-space samples^14^. Compressed SENSE takes advantage of both CS and the parallel imaging technique of sensitivity encoding (SENSE), and has been shown to accelerate image acquisition for several MRI applications^15–18^. However, compressed SENSE usually is applied to non-EPI scans^19^. Initial studies have recently shown, that a combination with EPI may reduce artifacts and improve image quality compared to standard SENSE DWI, making further image acquisition acceleration feasible^19,20^.

Therefore, the aim of this single-center, prospective study was to evaluate an accelerated DWI sequence of the prostate using echo planar imaging with compressed SENSE based reconstruction (EPICS, SmartSpeed Diffusion, Philips Healthcare, further referred to as DWI_EPICS_) in comparison to DWI with conventional SENSE (DWI_conv_) in a clinical setting.

## Methods

The local institutional review board (Ethics Committee of the Medical Faculty of the University of Bonn) approved this prospective study. The study was conducted in accordance with the Declaration of Helsinki and its amendments. All participants gave written informed consent prior to the examination. Patients could be included into the study when they were planned to undergo an MRI of the prostate due to clinical suspicion of prostate cancer (clinical suspicion based on results of digital rectal examination, serum prostate-specific antigen and/or transrectal ultrasound). Patients with contradictions for MRI such as claustrophobia, metallic implants, cardiac pacemakers and neurostimulators were excluded. MRI scans were acquired between August 2021 and January 2022.

### Imaging protocol

Participants underwent MRI acquisition on a 3 Tesla MRI scanner (3T Ingenia Elition X; Philips Healthcare; gradient system: 45 mT/m maximum amplitude, 220 T/m/s maximum slew rate; dual-source RF transmission technology) using a 28-channel anterior coil with a digital interface for signal reception. The routine prostate MRI protocol at our institution contains axial DWI with ADC maps generated from b-values of 100, 400 and 800 s/mm^2^ as well as an axial DWI with b = 1500s/mm². Furthermore, the protocol contains sagittal, coronal und axial SENSE accelerated T2 images with propeller acquisition (MultiVane XD), axial T1-weighted TSE images prior and after intravenous contrast media application with subtraction and axial dynamic-enhanced images. In addition to these routine protocol corresponding DWI sequences with EPICS were acquired in each patient: A 3 b-value scan (b = 100, 400, 800s/mm²) for ADC calculation and a DWI sequence with b = 1500s/mm. The technical parameters of the compared sequences are given in Table 1.

**Table 1:** Imaging parameters of the acquired conventional diffusion-weighted imaging (DWI) sequences and accelerated DWI sequences of the prostate using echo planar imaging and compressed SENSE based reconstruction (EPICS).

Sequence	3-b DWI	3-b EPICS	b1500 DWI	b1500 EPICS
b-value, s/mm^2^	100, 400, 800	100, 400, 800	1500	1500
Averages	2, 4, 8	2, 2, 4	18	10
TR, ms	5000	5000	3864	3914
TE	62	62	63	63
Matrix	80 × 76	80 × 76	80 × 76	80 × 76
Flip angle, degrees	90	90	90	90
Field of view, mm^2^	180 × 180	180 × 180	180 × 180	180 × 180
In-plane resolution acquired, mm	2.25/2.37/3.00	2.25/2.35/3.00	2.25/2.37/3.00	2.25/2.35/3.00
In-plane resolution reconstructed, mm	0.94/0.94/3.00	0.94/0.94/3.00	0.94/0.94/3.00	0.94/0.94/3.00
Slices	32	32	32	32
Slice thickness, mm	3	3	3	3
Acquisition time, s	215	125	250	139
SENSE-factor	2	2	2	2

*TR* repetition time, *TE* echo time, *s* seconds, *ms* milliseconds, *mm* millimeters.

### Quantitative image analysis

Quantitative image assessment of image intensities and ADC-values was performed by a radiologist (Y.C.L., with 2 years of experience) blinded to clinical patient data. Circular regions-of-interest (ROIs) were placed in prostate lesions if categorized as PIRADS 3 or above (in both peripheral and transitional zone) as well as in normal appearing peripheral zone of the prostate on all acquired DWI images and calculated ACD parameter maps (labeled as “lesion” and “PZ”). ROIs were drawn as large as possible on DWI with b = 800s/mm², excluding areas close to the rim of the lesion / the prostate to avoid partial-volume effects. After the anatomical position was cross-checked for pixel misalignments between different b-values, the ROI was copied to the other b-value images as well as to the related parameter map. Mean and standard deviation values were determined for each ROI.

The measured mean signal intensity (SI) with standard deviation (SD) of lesions (L) and normal peripheral zone of the prostate (PZ) were used to calculate:

- the apparent contrast-to-noise ratio: aCNR= (SI_L_ - SI_PZ_)/ SD_PZ_ and
- the apparent signal-to-noise ratio: aSNR = SI_L_ / SD_PZ_.

For patients without prostate lesions aSNR was calculated separately (aSNR = SI_PZ_ / SD_PZ_).

Mean ADC and coefficient of variation (CV) were compared between DWI_EPICS_ and DWI_conv_ to account for dispersion.

### Qualitative image analysis

Two radiologists with two (Y.C.L.) and eleven (C.C.P.) years of experience in prostate MRI evaluated DWI_conv_ and DWI_EPICS_ images independently regarding artifact extent of the prostate tissue, overall image quality and if applicable lesion conspicuity. Readers used a five-point-Likert scale for each criterion. Grades for artifacts were defined as follows: (1; non-diagnostic) excessive artifacts; (2; poor) pronounced artifacts; (3; intermediate) moderate artifacts; (4; good) minor artifacts; and (5; excellent) no artifacts. For lesion conspicuity and overall image quality following rating was defined: (1; non-diagnostic) highly restricted interpretability; (2; poor) restricted diagnostic interpretability; (3; intermediate) moderate diagnostic interpretability; (4; good) minor restrictions on diagnostic interpretability; and (5; excellent) unrestricted diagnostic interpretability. For assessment of tumor characterization in prostate MRI, strong diffusion weighting is important. Therefore, the high b-values 800 and 1500 were rated^21^. Additionally, the Prostate Imaging Reporting and Data System (PIRADS 2.1)^5^ was applied on both DWI_EPICS_ and DWI_conv_ separately. Both readers were blinded to clinical data and rated the acquired sequences in random order.

### Statistical analysis

Statistical analyses were conducted using IBM SPSS Version 27 (IBM Corp). Quantitative results are given as mean and standard deviation. Qualitative results are expressed as median with interquartile range (IQR). Wilcoxon signed-rank tests or paired sample t-tests were used for statistical analysis. Interrater reliability was assessed using the intraclass correlation coefficient (ICC). ICC estimates and their 95% confident intervals (CI) were calculated based on a mean-rating (k = 2), consistency, two-way mixed-effects model. P-values below 0.05 were considered significant.

## Results

### Participant characteristics

Between August 2021 and January 2022 36 male patients with clinically suspected prostate cancer were included in the study. One patient had to be excluded due to very pronounced motion artifacts on all acquired sequences. Overall, 35 patients were included into final data analysis with a mean age of 67 ± 8 years (range 52–81 years). 17/35 patients (48.6%) showed PIRADS 3 lesions or above of which 11/17 (64.7%) were histologically confirmed prostate carcinomas (Gleason 6: *n* = 4, Gleason 7: *n* = 5, Gleason 8: *n* = 1, Gleason 9: *n* = 1).

The average acquisition time of the 3-b-value DWI_EPICS_-sequence was 42% shorter than that of the DWI_conv_-sequence (125 vs. 215 s; *p* < 0.001) and average acquisition time of the DWI_EPICS_ b1500-sequence was 44% shorter than that of DWI_conv_ b1500-sequence (139 vs. 250 s; *p* < 0.001), leading to a total scan time reduction of DWI of approximately 43%. The overall scan time of the prostate MRI performed in accordance with PIRADS 2.1 including axial and sagittal T2 weighted sequences, axial T1 and axial Dynamic Contrast-Enhanced (DCE) sequences was 1143 s using DWI_conv_ compared to 942 s using DWI_EPICS_.

### Quantitative image analysis

**Fig. 1:**
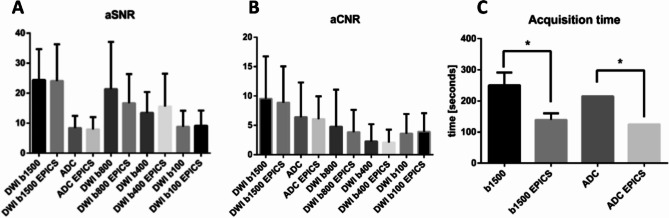
Mean overall apparent signal-to-noise ratio (aSNR) (**A**), and apparent contrast-to-noise ratio (aCNR) (**B**) and corresponding standard deviation within defined regions of interest plotted for conventional diffusion-weighted images (DWI) and accelerated DWI sequence of the prostate using echo planar imaging and compressed SENSE based reconstruction (EPICS). C shows acquisition times of the b1500 values scan and the 3b- value apparent diffusion coefficient (ADC) scan and corresponding standard deviation. P-values below 0.05 are marked with an asterisk.

Detailed quantitative results are given in Table 2; Fig. 1. Quantitative analysis revealed for both sequences no significant differences between parameters obtained conventionally and with EPICS, neither for aCNR_Lesion_, and aSNR_Lesion_ nor for aSNR_PZ_. A non-significant tendency towards higher aSNR_Lesion_ values on DWI_EPICS_ was found for b400 (15.59 ± 10.89 vs. 13.45 ± 6.92, *p* = 0.08) and b100 values (9.11 ± 5.06 vs. 8.88 ± 5.33, *p* = 0.81). For b800 in patients with prostate lesions aCNR_Lesion_ (3.85 ± 3.79 vs. 4.77 ± 6.30, *p* = 0.94) and aSNR_Lesion_ (16.69 ± 9.70 vs. 21.39 ± 15.7; *p* = 0.41) were slightly lower for DWI_EPICS_, whereas for b400 and b100 aCNR_Lesion_ (b400: 2.28 ± 2.91 vs. 2.07 ± 2.20, *p* = 0.98; b100: 3.91 ± 3.16 vs. 3.60 ± 3.32, *p* = 0.60) were slightly higher for DWI_EPICS_ compared to DWI_conv_. DWI_EPICS_ yielded a slightly higher aCNR (9.53 ± 7.22 vs. 8.88 ± 6.16 *p* = 0.55) and aSNR (24.37 ± 10.28 vs. 24.08 ± 12.2, *p* = 0.98) for the b1500-sequence.

**Table 2:** Results of apparent signal-to-noise ratio (aSNR) and apparent contrast-to-noise ratio (aCNR) analysis for patients with and without prostate lesions. Moreover, p-values of the comparison between conventional diffusion-weighted imaging (DWI) and accelerated DWI sequence of the prostate using echo planar imaging and compressed SENSE based reconstruction (EPICS) were given. P-values below 0.05 were considered significant. PZ: Peripheral zone.

	b1500 conv	b1500 EPICS	*p*-value	b800 conv	b800 EPICS	*p*-value	b400 conv	b400 EPICS	*p*-value	b100 conv	b100 EPICS	*p*-value
aSNR_Lesion_ (*n* = 17)	24.37 ± 10.28	24.08 ± 12.2	0.98	21.39 ± 15.70	16.69 ± 9.70	0.41	13.45 ± 6.92	15.59 ± 10.89	0.08	8.82 ± 5.33	9.11 ± 5.06	0.81
aCNR_Lesion_ (*n* = 17)	9.53 ± 7.22	8.88 ± 6.16	0.55	4.77 ± 6.30	3.85 ± 3.79	0.94	2.28 ± 2.91	2.07 ± 2.20	0.98	3.60 ± 3.32	3.91 ± 3.16	0.60
aSNR_PZ_ (*n* = 18)	15.18 ± 6.48	15 ± 7.4	0.94	17.03 ± 10.96	13.7 ± 6.56	0.35	14.08 ± 7.57	16.04 ± 9.36	0.17	12.71 ± 8.25	12.05 ± 5.98	0.88

Mean ADC (b100, b400, b800) and CV of ADC of the peripheral zone of the prostate (ADC: 1.74 ± 0.16 vs. 1.75 ± 0.14; *p* = 0.87; CV of ADC: 0.09 ± 0.02 vs. 0.08 ± 0.04; *p* = 0.41) and of the lesions (ADC: 1.07 ± 0.14 vs. 1.06 ± 0.10; *p* = 0.83; CV of ADC: 0.13 ± 0.06 vs. 0.10 ± 0.05; *p* = 0.13) also showed no significant differences for DWI_conv_ and DWI_EPICS_ (Table 3).

**Table 3:** Results of apparent diffusion coefficient (ADC) and ADC coefficient of variation (CV) analysis in prostate and lesions for diffusion-weighted imaging (DWI) and accelerated DWI sequence of the prostate using echo planar imaging and compressed SENSE based reconstruction (EPICS).

	ADC			CV of ADC		
	DWI conv	DWI EPICS	*p*-value	DWI conv	DWI EPICS	*p*-value
Peripheral zone (*n* = 18)	1.74 ± 0.16	1.75 ± 0.14	0.87	0.09 ± 0.02	0.08 ± 0.04	0.41
Lesion (*n* = 17)	1.07 ± 0.14	1.06 ± 0.10	0.83	0.13 ± 0.06	0.10 ± 0.05	0.13

Given are mean values ± standard deviation together with corresponding p-values. P-values below 0.05 were considered significant.

### Qualitative image analysis

**Fig. 2:**
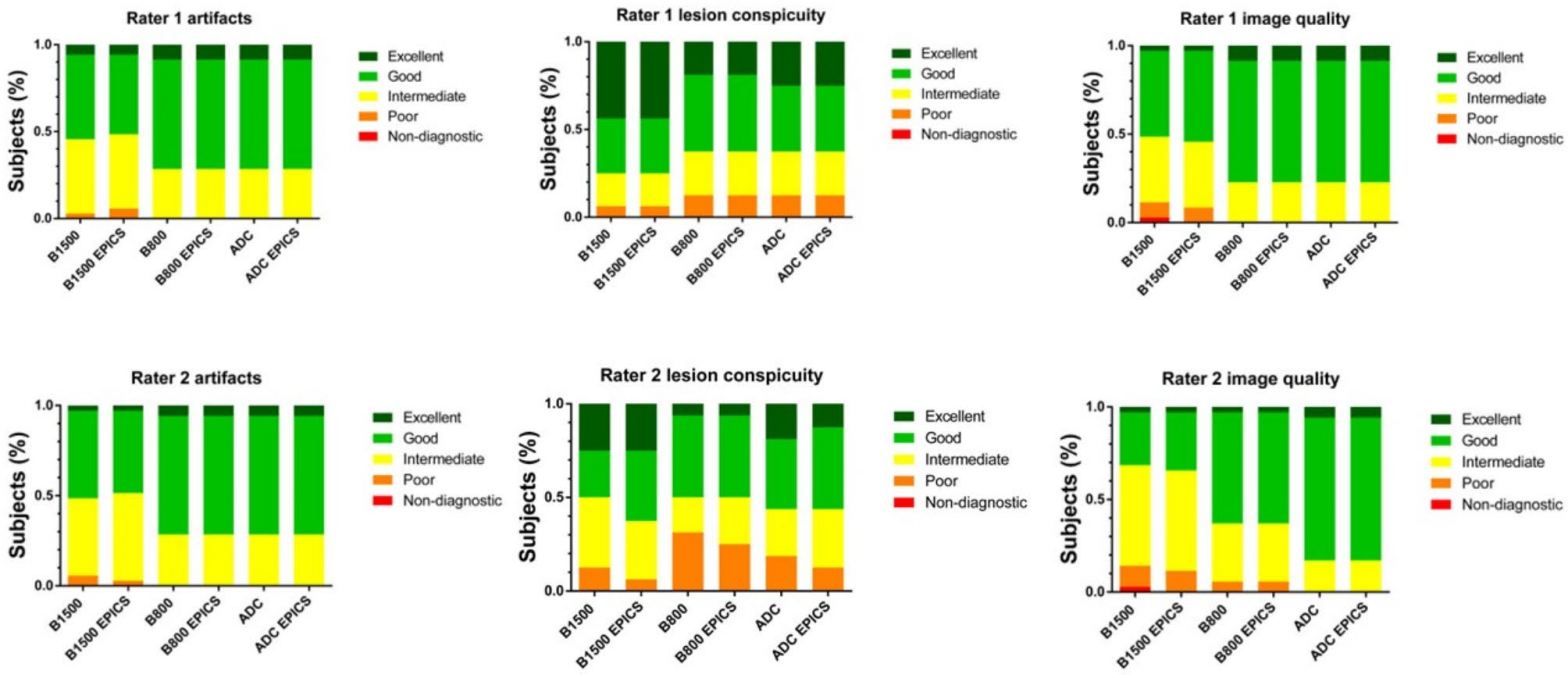
Bar plots show ratings of artifacts, lesion conspicuity and overall image quality for conventional diffusion-weighted images (DWI) and accelerated DWI sequence using echo planar imaging and compressed SENSE based reconstruction (EPICS) with b1500, b800 and apparent diffusion coefficient (ADC).

**Fig. 3:**
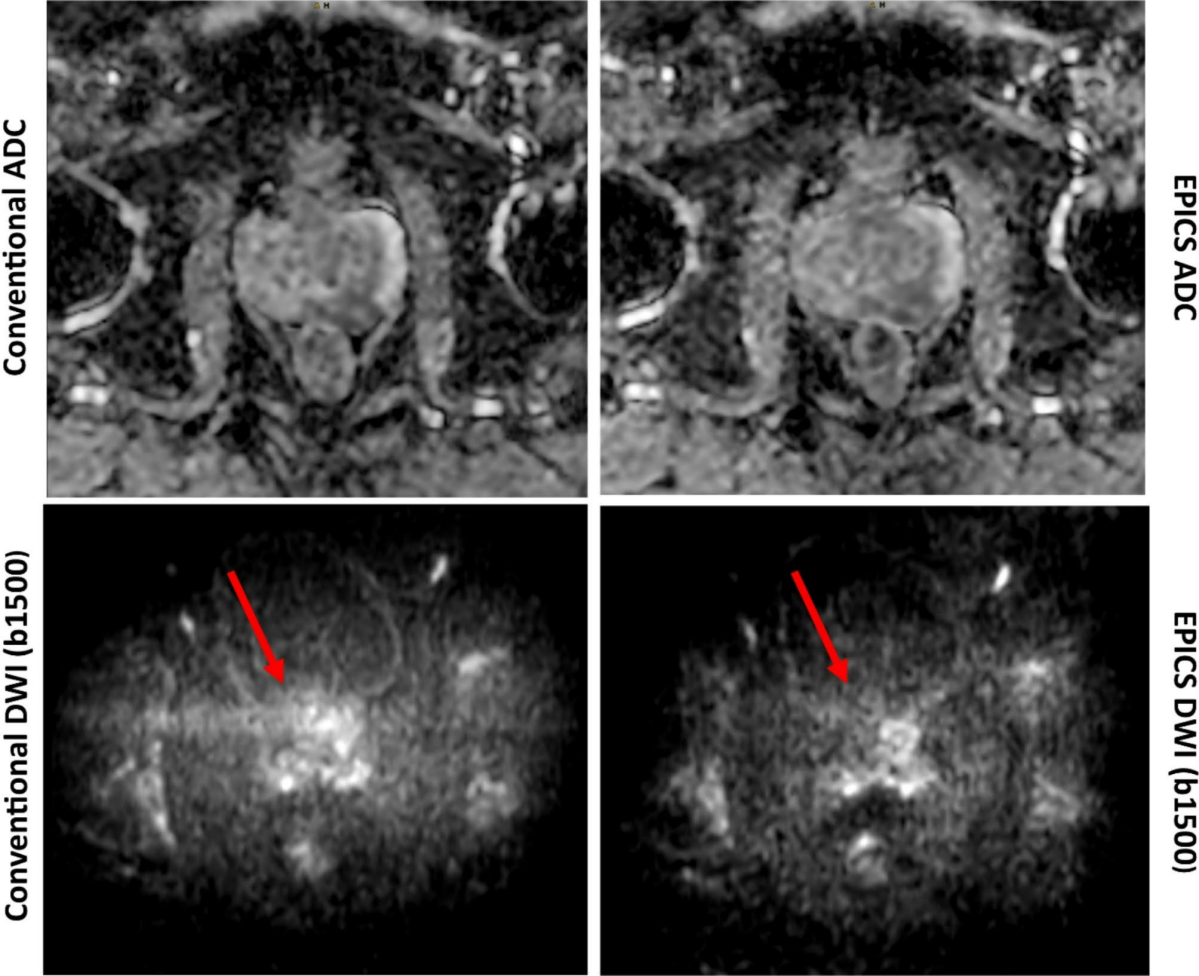
DWI b1500 images and apparent diffusion coefficient (ADC) maps obtained from conventional diffusion-weighted images (DWI) and accelerated DWI sequences using echo planar imaging and compressed SENSE based reconstruction (EPICS). In this example artifacts (arrows) are reduced in EPICS DWI (b1500) compared to conventional reconstruction. No significant changes are seen in the ADC maps.

There was no significant difference in the rating of artifacts, lesion conspicuity and overall image quality between DWI_conv_ and DWI_EPICS_ (Table 4; Fig. 2). Only minor differences were noted in image quality with scores ranging from 2 to 5 for DWI_EPICS_ (b1500) and 1–5 for DWI_conv_ (b1500). Overall artifacts were rated with a median of 3.5 for DWI_EPICS_ in b1500 images and 4.0 for DWI_conv_, however remaining non-significant (*p* = 0.77). In two cases artifacts were remarkably reduced for DWI_EPICS_ compared to DWI_conv_ (Fig. 3).

**Table 4:** Results of the ratings on a 5-point-Likert-scale from 1–5 (non-diagnostic - excellent) for artifacts, lesion conspicuity and overall image quality of diffusion-weighted imaging (DWI) and accelerated DWI sequence of the prostate using echo planar imaging and compressed SENSE based reconstruction (EPICS) with b1500, b800 and apparent diffusion coefficient (ADC).

	b1500 conv	b1500 EPICS	*p*-value	b800 conv	b800 EPICS	*p*-value	ADC conv	ADC EPICS	*p*-value
Artifacts (*n* = 35)	4 (2–5)	3.5 (2–5)	0.77	4 (3–5)	4 (3–5)	1	4 (3–5)	4 (3–5)	1
Lesion conspicuity (*n* = 17)	4 (2–5)	4 (2–5)	0.25	4 (2–5)	4 (2–5)	0.99	4 (2–5)	4 (2–5)	0.99
Image quality (*n* = 35)	3 (1–5)	3 (2–5)	0.11	4 (3–5)	4 (3–5)	1	4 (3–5)	4 (3–5)	1

Given are median values and range together with corresponding p-values. P-values below 0.05 were considered significant.

PIRADS-scores of each patient did not differ between DWI_conv_ and DWI_EPICS_ (ICC = 1). 5.7% (2/35) of the patients were rated as PIRADS 1, 48.6% (17/35) as PIRADS 2, 11.4% (4/35) as PIRADS 3, 17.1% (6/35) as PIRADS 4 and 17.1% (6/35) as PIRADS 5. Interobserver reliability for overall qualitative scoring was good (ICC = 0.82; (95% CI: 0.786; 0.849)), with an ICC of 0.916 (95% CI: 0.890; 0.936) for artifacts, an ICC of 0.826 (95% CI: 0.772; 0.867) for image quality, and an ICC of 0.737 (95% CI: 0.605; 0.824) for lesion conspicuity.

## Discussion

The aim of this single-center, prospective study was to evaluate an accelerated DWI sequence of the prostate using echo planar imaging with compressed SENSE based reconstruction and assess its performance in comparison to conventional DWI in patients suspected of prostate cancer. The evaluated sequences considerably reduced the acquisition time up to 44% and achieved overall good image quality comparable to the conventional DWI sequence.

Acceleration of image acquisition is of major concern due to the constantly increasing demand for MRI examinations of the prostate^22,23^. There are various approaches for the acceleration of image acquisition, e.g. 3D T2WI opposed to the usual multiplanar 2D T2 weighted images, acquisition of 2D TSE using radial acquisition, improvement of through-plane resolution, and adjusted acquisition parameters combined with AI-based reconstruction techniques^9,24–33^. All of these techniques focus on the T2 sequences which account for approximately 60% of the acquisition time^22^. Yet, there also is a need for further improvement of the DWI, which is the dominant sequence for prostate cancer of the peripheral zone of the prostate. The evaluated EPICS technique addresses this issue and accelerates the DWI using an echo planar imaging technique combined with compressed SENSE. EPICS is based on single-shot DW-EPI acquisition. Its sampling pattern was not modified and the Compressed SENSE framework was applied for reconstruction. The employed technique utilizes a regular SENSE undersampling (acceleration factor of 2 for both scans DWI_conv_ and DWI_EPICS_) with a reconstruction using an iterative SENSE algorithm with L1 norm regularization with a wavelet sparsifying transform similar to non-EPI compressed SENSE reconstructions^34^. Acceleration of the DWI_EPICS_ was achieved by reducing the b-value specific number of averages (Table 1).

The scan time reduction optimizes patient`s convenience lowering the likelihood of motion artifacts and therefore benefits care of patients with suspected prostate cancer. Additionally patient through-put can be increased and resources can be optimized, enabling reduced costs in the healthcare system and shorter waiting times for examinations.

**Fig. 4:**
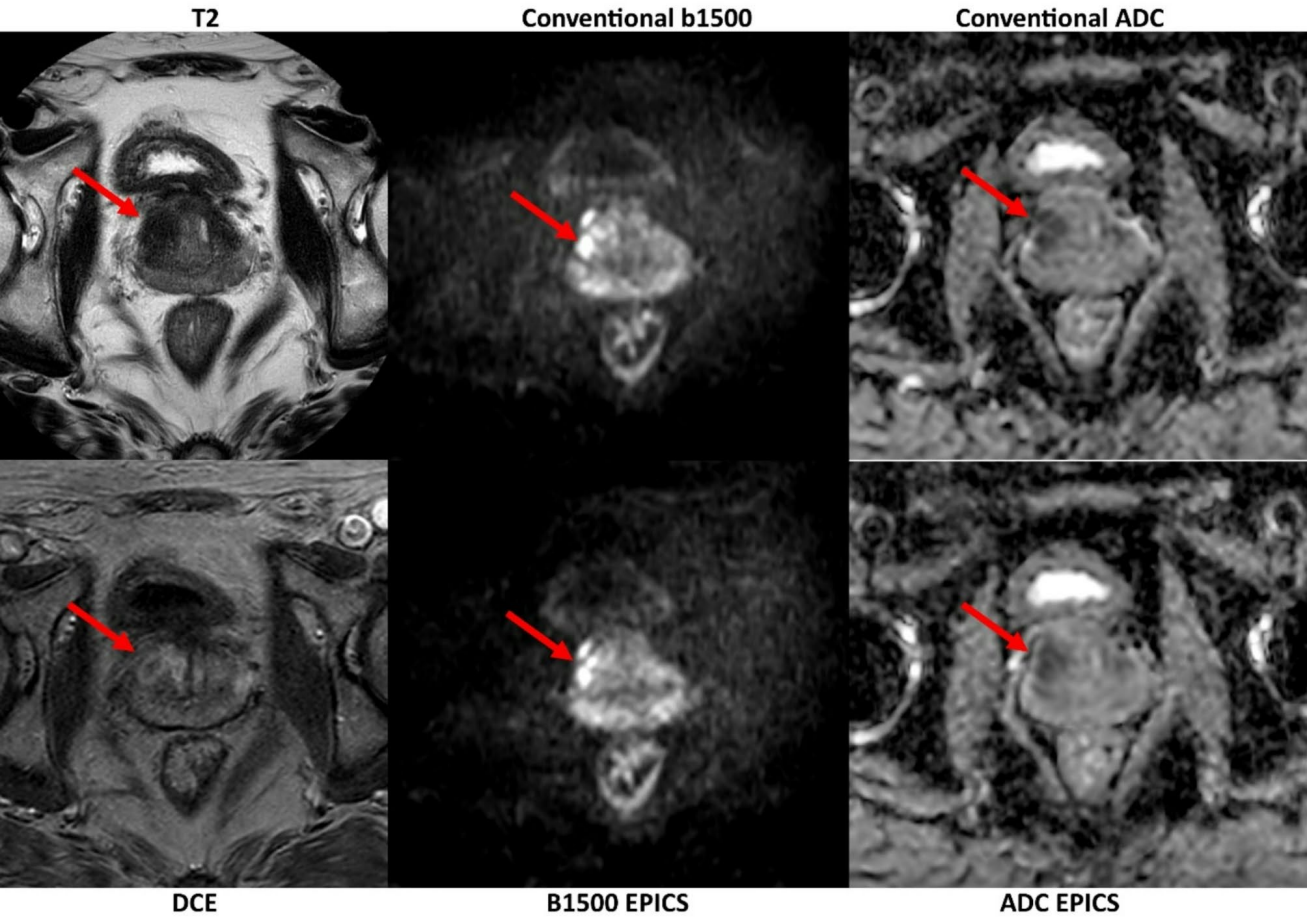
T2 image, dynamic contrast-enhanced (DCE) image, as well as DWI b1500 images and apparent diffusion coefficient (ADC) maps obtained from conventional diffusion-weighted images (DWI) and accelerated DWI sequences using echo planar imaging and compressed SENSE based reconstruction (EPICS). A PIRADS 5 lesion is shown in the right mid anterior transitional zone (red arrows).

As a negative correlation between Gleason score and ADC has been reported consistently in multiple studies, and therefore ADC is an important marker of prostate carcinoma, consistency in ADC measurements are important^35–37^. In this study image quality of ADC and ADC values of EPICS have not shown significant deviation from conventional ADC (Fig. 4). In some cases artifacts were reduced in DWI_EPICS_-sequences whereas others showed marginally increased artifacts. In a few cases a reduction of “noise-band” artifacts, which are amplified in particular by many averages as seen in Fig. 3 was observed. These artifacts are dependent on patient size and not consistent throughout the observed patients. Due to the underlying technique other artifacts were not expected to show differences and no significant differences were found (see Table 5). It cannot be ruled out that artifacts may also have changed due to minimal movements between the scan of the standard and research sequences. However, these did not limit image quality or image interpretation.

**Table 5:** Results of the ratings on a 5-point-Likert-scale from 1–5 (non-diagnostic - excellent) for different artifacts of diffusion-weighted imaging (DWI) and accelerated DWI sequence of the prostate using echo planar imaging and compressed SENSE based reconstruction (EPICS).

	DWI conv	DWI epics	*p*-value
Geometric distortion	4 (3–5)	4 (3–5)	0.99
Signal loss	4 (3–5)	4 (3–5)	0.82
Motion artifacts	3 (2–5)	3 (2–5)	0.76
Residual aliasing	4 (3–5)	4 (3–5)	0.99
Noise-band artifacts	3 (2–5)	3.5 (3–5)	0.24

Given are median values and range together with corresponding p-values. P-values below 0.05 were considered significant.

**Fig. 5:**
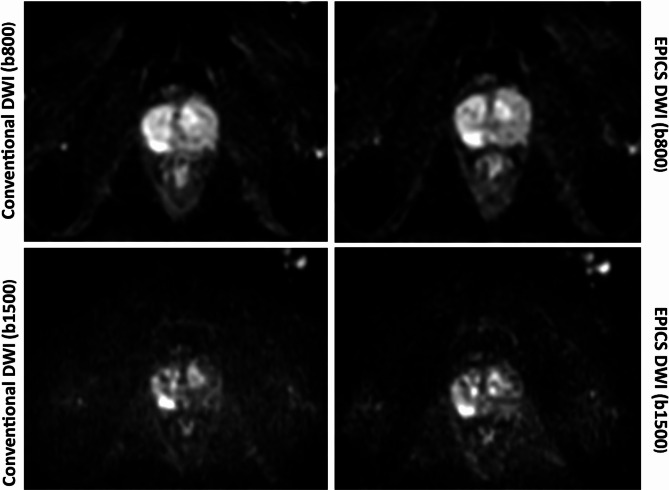
b800 and b1500 images obtained from conventional diffusion-weighted images (DWI) and accelerated DWI sequences using echo planar imaging and compressed SENSE based reconstruction (EPICS) illustrating the variability in perceptual assessment of the readers. Reader 2 rated lesion conspicuity in b800_conv_ and b1500_conv_ a 3 compared to a 4 in b800_EPICS_ and b1500_EPICS_. Reader 1 rated all images with a 4.

In rare cases non-significant differences between readers were found as shown in Fig. 5, showcasing one representative case where differences in lesion conspicuity were noted between raters.

As the PIRADS-score was identical between both techniques in all patients, the acquisition technique had no adverse impact on clinical decisions. Therefore, the evaluated diffusion-weighted imaging sequence with EPICS offers a potential to accelerate diffusion-weighted images in MRI without an effect on image interpretation.

The study showed that for some b-values EPICS showed a better quantitative performance, whereas for others DWI_conv_ was superior. The differences in aSNR and aCNR for the b-values can be explained by the different impact of the averaging of each b-value. Therefore it could be feasible to differentiate the averaging for each of the b-values to optimize image quality. Aim of the study was an acceleration of DWI-sequences in the prostate in real patients suspected for prostate cancer at comparable image quality, therefore it was decided to implement the averaging as stated in Table 1 based on prior experiences. Testing further possible different settings was not feasible due to the significantly longer total scan time of the study scan in older and mostly sick patients.

An alternative approach might be to reinvest the time saved in order to improve image quality with the same number of averages and thus same acquisition time. This idea was not investigated in the present study and could be a promising approach to further improve the quality of prostate MRI and in particular to further increase the precision of MRI-fused biopsies. However this has been focus of the other, presently rare, clinical studies on DWI_EPICS_. All of these studies focused primarily on improvement of image quality instead of acceleration of acquisition time. For multiparametric prostate MRI, Tamada et al. reported improved image quality and comparable diagnostic performance for equal acquisition times compared to DWI with single-shot EPI using SENSE^20^. Yoneyama et al. showed a noise reduction in prostate DWI using EPICS^38^. For abdominal imaging Kaga et al. reported significantly improved image quality and higher ADC values compared with conventional single-shot EPI with PI^19^. This was also confirmed for head and neck diffusion-weighted imaging in a comparative study with fixed acquisition times^39^. In another study with fixed acquisition times, improved conspicuity of liver lesions in DWI_EPICS_ of the abdomen was found^40^. In brain imaging of the cerebral cortex a high reproducibility of DWI_EPICS_ was stated recently^27^.

There are many approaches to shorten MRI acquisition times of the prostate. Most recent studies focus on Deep Learning based methods^28,41–43^.The combination of these Deep Learning approaches with EPICS could potentially further accelerate image acquisition. Further studies should focus on possible combinations of these techniques.

Several limitations apply to our study: First, DWI sequence parameters have not been optimized, therefore further optimization to find the best mix of acquisition acceleration and image quality for clinical use is necessary. Phantom studies could help to find ideal settings and detect deviations in the sequences and should be part of further studies. However, a limitation of phantom studies would have been, that the image content is crucial for imaging quality. Therefore, clinical applicability is only possible to a limited extent. Second, we only investigated diffusion-weighted images, even though for PIRADS 2.1 multiparametric MRI is used. As all clinically necessary sequences were acquired, the readers were able to use them for image orientation and lesion classification. Third, the study design was single-centered, investigating only a small group of patients examined on one single 3.0-T MRI system, which could have caused a selection bias. Even though no differences in the PIRADS-scores were found, these could occur in a larger patient group. Further, ideally multicentric, studies on a larger patient cohort are needed to show the full potential of the sequences in clinical routine. Lastly, not every participant underwent biopsy as the decision for biopsy was made by the referring urologists, therefore a precise correlation of PIRADS and histopathological findings was not possible.

In conclusion, the evaluated accelerated diffusion-weighted imaging sequence using echo planar imaging with compressed SENSE based reconstruction significantly reduced the acquisition time by 43% with maintaining diagnostic quality compared to the conventional sequence. The reduced scan time allows for a considerable increase of the overall number of scanned patients, increased patient convenience, less potential of motion artifacts and therefore benefits care of patients.

## Data Availability

The anonymized datasets generated during and analyzed during the current study are available from the corresponding author on reasonable request.

## References

[1] Weiss, J. et al. Implementation of a 5-Minute magnetic resonance imaging screening protocol for prostate cancer in men with elevated prostate-Specific antigen before biopsy. *Invest. Radiol.* **53** (3), 186–190 (2018). 29077588 10.1097/RLI.0000000000000427

[2] Hoeks, C. M. A. et al. Prostate cancer: multiparametric MR imaging for detection, localization, and staging. *Radiology* **261** (1), 46–66 (2011). 21931141 10.1148/radiol.11091822

[3] Purysko, A. S. et al. Accuracy and Interobserver Agreement for Prostate Imaging Reporting and Data System. Version 2, for the Characterization of Lesions Identified on Multiparametric MRI of the Prostate. *AJR Am. J. Roentgenol.* **209** (2), 339–349 (2017). 10.2214/AJR.16.17289 28570099

[4] Scheenen, T. W. J., Rosenkrantz, A. B., Haider, M. A. & Fütterer, J. J. Multiparametric magnetic resonance imaging in prostate cancer management: current status and future perspectives. *Invest. Radiol.* **50** (9), 594–600 (2015). 25974203 10.1097/RLI.0000000000000163

[5] Turkbey, B. et al. Prostate Imaging Reporting and Data System Version 2.1: 2019 Update of Prostate Imaging Reporting and Data System Version 2. *Eur. Urol.* **76**(3), 340–351 (2019). 10.1016/j.eururo.2019.02.033 30898406

[6] Yang, L., Li, M., Zhang, M-N., Yao, J. & Song, B. Association of prostate zonal volume with location and aggressiveness of clinically significant prostate cancer: A multiparametric MRI study according to PI-RADS version 2.1. *Eur. J. Radiol.* **150**, 110268 (2022). 35344914 10.1016/j.ejrad.2022.110268

[7] Tamada, T. et al. Diffusion-weighted imaging in prostate cancer. *MAGMA* **35** (4), 533–547 (2022). 34491467 10.1007/s10334-021-00957-6

[8] Pernar, C. H., Ebot, E. M., Wilson, K. M. & Mucci, L. A. The epidemiology of prostate cancer. *Cold Spring Harb Perspect. Med.* **8**(12). (2018). 10.1101/cshperspect.a030361 PMC6280714 29311132

[9] Bischoff, L. M. et al. T2 turbo spin echo with compressed sensing and propeller acquisition (Sampling k-Space by utilizing rotating Blades) for fast and motion robust prostate MRI: comparison with conventional acquisition. *Invest. Radiol.*10.1097/RLI.0000000000000923 (2022). 36070533 10.1097/RLI.0000000000000923

[10] Hennig, J., Nauerth, A. & Friedburg, H. RARE imaging: a fast imaging method for clinical MR. *Magn. Reson. Med.* **3** (6), 823–833 (1986). 3821461 10.1002/mrm.1910030602

[11] Mansfield, P. Real-time echo-planar imaging by NMR. *Br. Med. Bull.* **40** (2), 187–190 (1984). 6744006 10.1093/oxfordjournals.bmb.a071970

[12] Frahm, J., Haase, A. & Matthaei, D. Rapid NMR imaging of dynamic processes using the FLASH technique. *Magn. Reson. Med.* **3** (2), 321–327 (1986). 3713496 10.1002/mrm.1910030217

[13] Jaspan, O. N., Fleysher, R. & Lipton, M. L. Compressed sensing MRI: a review of the clinical literature. *Br. J. Radiol.* **88** (1056), 20150487 (2015). 26402216 10.1259/bjr.20150487 PMC4984938

[14] Feng, L. et al. Compressed sensing for body MRI. *J. Magn. Reson. Imaging*. **45** (4), 966–987 (2017). 27981664 10.1002/jmri.25547 PMC5352490

[15] Yu, V. Y. et al. Combined compressed sensing and SENSE to enhance radiation therapy magnetic resonance imaging simulation. *Adv. Radiat. Oncol.* **7** (1), 100799 (2022). 34765805 10.1016/j.adro.2021.100799 PMC8569477

[16] Wang, M. et al. Acceleration of pCASL-Based cerebral 4D MR angiography using compressed SENSE: A comparison with SENSE. *Front. Neurol.* **13**, 796271 (2022). 35386411 10.3389/fneur.2022.796271 PMC8977489

[17] Meister, R. L. et al. Compressed SENSE in pediatric brain tumor MR imaging assessment of image quality, examination time and energy release. *Clin. Neuroradiol.* **32** (3), 725–733 (2022). 34994810 10.1007/s00062-021-01112-3 PMC9424145

[18] Gong, X. et al. Comparison of compressed sensing-sensitivity encoding (CS-SENSE) accelerated 3D T2W TSE sequence versus conventional 3D and 2D T2W TSE sequences in rectal cancer: a prospective study. *Abdom. Radiol. (NY)*. **47** (11), 3660–3670 (2022). 35997800 10.1007/s00261-022-03636-9 PMC9560929

[19] Kaga, T. et al. Diffusion-weighted imaging of the abdomen using echo planar imaging with compressed SENSE: feasibility, image quality, and ADC value evaluation. *Eur. J. Radiol.* **142**, 109889 (2021). 34388627 10.1016/j.ejrad.2021.109889

[20] Tamada, T. et al. Clinical application of single-shot echo-planar diffusion-weighted imaging with compressed SENSE in prostate MRI at 3T: preliminary experience. *MAGMA* **35** (4), 549–556 (2022). 35403993 10.1007/s10334-022-01010-w

[21] A R, M J TB, N. C., Gh, M. S. M., Gh, H. & A Signal intensity of high B-value Diffusion-weighted imaging for the detection of prostate cancer. *J. Biomed. Phys. Eng.* **9** (4), 453–458 (2019). 31531298 10.31661/jbpe.v0i0.811 PMC6709361

[22] Mir, N., Fransen, S. J., Wolterink, J. M., Fütterer, J. J. & Simonis, F. F. J. Recent developments in speeding up prostate MRI. *J. Magn. Reson. Imaging*. 10.1002/jmri.29108 (2023). 37982353 10.1002/jmri.29108

[23] Oberlin, D. T., Casalino, D. D., Miller, F. H. & Meeks, J. J. Dramatic increase in the utilization of multiparametric magnetic resonance imaging for detection and management of prostate cancer. *Abdom. Radiol. (NY)*. **42** (4), 1255–1258 (2017). 27858090 10.1007/s00261-016-0975-5

[24] Choi, M. H., Lee, Y. J., Jung, S. E. & Han, D. High-resolution 3D T2-weighted SPACE sequence with compressed sensing for the prostate gland: diagnostic performance in comparison with conventional T2-weighted images. *Abdom. Radiol. (NY)*. **48** (3), 1090–1099 (2023). 36544053 10.1007/s00261-022-03777-x

[25] Polanec, S. H. et al. 3D T2-weighted imaging to shorten multiparametric prostate MRI protocols. *Eur. Radiol.* **28** (4), 1634–1641 (2018). 29134351 10.1007/s00330-017-5120-5 PMC5834556

[26] Meier-Schroers, M. et al. Revised PROPELLER for T2-weighted imaging of the prostate at 3 Tesla: impact on lesion detection and PI-RADS classification. *Eur. Radiol.* **28** (1), 24–30 (2018). 28687915 10.1007/s00330-017-4949-y

[27] Yamashita, K. et al. Reproducibility of quantitative ADC, T1, and T2 measurement on the cerebral cortex: utility of whole brain echo-planar DWI with compressed SENSE (EPICS-DWI): A pilot study. *Eur. J. Radiol. Open.* **11**, 100516 (2023). 37609044 10.1016/j.ejro.2023.100516 PMC10440392

[28] Bischoff, L. M. et al. Deep learning Super-Resolution reconstruction for fast and Motion-Robust T2-weighted prostate MRI. *Radiology* **308** (3), e230427 (2023). 37750774 10.1148/radiol.230427

[29] Gassenmaier, S. et al. Accelerated T2-Weighted TSE Imaging of the Prostate Using Deep Learning Image Reconstruction: A Prospective Comparison with Standard T2-Weighted TSE Imaging. *Cancers (Basel)* **13**(14) (2021). 10.3390/cancers13143593 PMC8303682 34298806

[30] Borisch, E. A. et al. Model-based image reconstruction with wavelet sparsity regularization for through-plane resolution restoration in T2 -weighted spin-echo prostate MRI. *Magn. Reson. Med.* **89** (1), 454–468 (2023). 36093998 10.1002/mrm.29447 PMC9617775

[31] Kargar, S. et al. Use of kZ -space for high through-plane resolution in multislice MRI: application to prostate. *Magn. Reson. Med.* **81** (6), 3691–3704 (2019). 30844092 10.1002/mrm.27691

[32] Kargar, S. et al. Modified acquisition strategy for reduced motion artifact in super resolution T2 FSE multislice MRI: application to prostate. *Magn. Reson. Med.* **84** (5), 2537–2550 (2020). 32419197 10.1002/mrm.28315 PMC7402017

[33] Harder, F. N. et al. Prospectively Accelerated T2-Weighted Imaging of the Prostate by Combining Compressed SENSE and Deep Learning in Patients with Histologically Proven Prostate Cancer. *Cancers (Basel)* **14**(23). (2022). 10.3390/cancers14235741 PMC9738899 36497223

[34] Kamal, O. et al. Noise reduction in diffusion weighted MRI of the pancreas using an L1-regularized iterative SENSE reconstruction. *Magn. Reson. Imaging*. **87**, 1–6 (2022). 34808306 10.1016/j.mri.2021.11.009

[35] Wang, X. et al. Comparison of single-scanner single-protocol quantitative ADC measurements to ADC ratios to detect clinically significant prostate cancer. *Eur. J. Radiol.* **136**, 109538 (2021). 33482592 10.1016/j.ejrad.2021.109538

[36] Falaschi, Z. et al. Non-timely clinically applicable ADC ratio in prostate MpMRI: a comparison with fusion biopsy results. *Abdom. Radiol. (NY)*. **47** (11), 3855–3867 (2022). 35943517 10.1007/s00261-022-03627-w PMC9560938

[37] Kitajima, K. et al. Do apparent diffusion coefficient (ADC) values obtained using high b-values with a 3-T MRI correlate better than a transrectal ultrasound (TRUS)-guided biopsy with true Gleason scores obtained from radical prostatectomy specimens for patients with prostate cancer? *Eur. J. Radiol.* **82** (8), 1219–1226 (2013). 23518144 10.1016/j.ejrad.2013.02.021

[38] Yoneyama, M., Morita, K., Peeters, J., Nakaura, T. & van Cauteren, M. Noise Reduction in Prostate Single-Shot DW-EPI utilizing Compressed SENSE Framework,. Proc. ISMRM. :1634. (2019).

[39] Yoshida, N. et al. Echo planar imaging with compressed sensitivity encoding (EPICS): usefulness for head and neck diffusion-weighted MRI. *Eur. J. Radiol.* **155**, 110489 (2022). 36037584 10.1016/j.ejrad.2022.110489

[40] Kaga, T. et al. Diagnostic ability of diffusion-weighted imaging using echo planar imaging with compressed SENSE (EPICS) for differentiating hepatic hemangioma and liver metastasis. *Eur. J. Radiol.* **167**, 111059 (2023). 37643558 10.1016/j.ejrad.2023.111059

[41] Tong, A. et al. Comparison of a deep Learning-Accelerated vs. Conventional T2-Weighted sequence in biparametric MRI of the prostate. *J. Magn. Reson. Imaging*. 10.1002/jmri.28602 (2023). 36651358 10.1002/jmri.28602 PMC10352465

[42] Gassenmaier, S. et al. Deep learning-accelerated T2-weighted imaging of the prostate: reduction of acquisition time and improvement of image quality. *Eur. J. Radiol.* **137**, 109600 (2021). 33610853 10.1016/j.ejrad.2021.109600

[43] Johnson, P. M. et al. Deep learning reconstruction enables highly accelerated biparametric MR imaging of the prostate. *J. Magn. Reson. Imaging*. **56** (1), 184–195 (2022). 34877735 10.1002/jmri.28024 PMC9170839

